# On the Chameleonic Behaviour of Cholesterol through a Fractal/Multifractal Model

**DOI:** 10.1155/2020/6217691

**Published:** 2020-01-06

**Authors:** Nicolae Dan Tesloianu, Vlad Ghizdovat, Maricel Agop, Cristina Rusu, Anca Cardoneanu

**Affiliations:** ^1^Cardiology Clinic, Clinical Emergency Hospital “Sf. Spiridon” Iasi, University of Medicine and Pharmacy “Gr. T. Popa”, 16 University Str., Iaşi 700115, Romania; ^2^“Grigore T. Popa” University of Medicine and Pharmacy, Faculty of Medicine, Biophysics and Medical Physics Department, 16 University Str., Iaşi 700115, Romania; ^3^Physics Department, “Gheorghe Asachi” Technical University, Prof. Dr. Docent Dimitrie Mangeron Rd., No. 59A, Iaşi 700050, Romania; ^4^Academy of Romanian Scientist, 54 Splaiul Independentei, Sector 5, Bucureşti 050094, Romania; ^5^“Grigore T. Popa” University of Medicine and Pharmacy, Faculty of Medicine, Rheumatology Department, 16 University Str., Iaşi 700115, Romania

## Abstract

An increasing number of studies are beginning to show that both low-density lipoprotein and high-density lipoprotein cholesterol can constitute risk factors for myocardial infarction. Such a behaviour has been called by experts in the field the “chameleonic effect” of cholesterol. In the present paper, a fractal/multifractal model for low-density lipoprotein and high-density lipoprotein cholesterol dynamics is proposed. In such a context, a fractal/multifractal tunneling effect for systems with spontaneous symmetry breaking is analyzed so that if the spontaneous symmetry breaking is assimilated to an inflammation (in the form of a specific scalar potential), then a coupling between two fractal/multifractal states can be observed. These two states, which have been associated to biological structures such as low-density lipoprotein and high-density lipoprotein, transfer their states through a fractal/multifractal tunneling effect. Moreover, in our opinion, the widely used notions of “good” and “bad” cholesterol must be redefined as two different states (low-density lipoprotein and high-density lipoprotein) of the same biological structure named “cholesterol.” In our work, for the first time in the specialized literature, low-density lipoprotein and high-density lipoprotein have been regarded as two different states of the same biological structure (named “cholesterol”), such as in nuclear physics, the neutron and proton are two different states of the same particle named nucleon.

## 1. Introduction: Current Evidence of the Chameleonic Behaviour of Cholesterol

Cholesterol fractions, especially low-density lipoprotein (LDL) and high-density lipoprotein (HDL) cholesterol, are frequently analyzed biomarkers in clinical laboratories [1]. We remind that lipoproteins, which are combinations of fats (lipids) and proteins, are the form in which lipids are transported in the blood, for details see [2, 3].

Observational studies have shown that LDL and HDL have opposing associations with risk of myocardial infarction, with LDL cholesterol being a positive factor and HDL cholesterol being a negative (protective) factor [2, 3].

However, in the following paragraphs we want to show, by doing a synopsis of the literature in this field that, in recent years, more and more evidence points to the fact that a chameleonic behavior can be attributed to LDL and HDL cholesterol.

Observational studies cannot separate the causal role in the pathological process from the role of a marker of the underlying pathophysiology. The results of both randomized trials of LDL cholesterol-lowering treatments [4] and from human Mendelian diseases [5, 6] are suggesting that plasma LDL cholesterol is related to risk of myocardial infarction. However, little evidence is available for the causal relevance of HDL cholesterol from randomized trials or Mendelian diseases, and the existing ones are usually not consistent [7, 8]. Moreover, more and more studies are starting to oppose the idea that raising of plasma HDL cholesterol will surely translate into a risk reduction of myocardial infarction [9].

If particular plasma biomarkers are directly involved in a pathological process, then inherited variation changing plasma concentrations of these biomarkers should affect the risk of disease in the direction and magnitude predicted by the plasma concentrations. This approach has been used in the past to analyze plasma HDL cholesterol; although with restricted sample sizes, a small number of single nucleotide polymorphisms (SNPs) at a few genes and with SNPs that affect multiple lipid fractions [10, 11].

This is a main reason for which current studies have not been able to completely resolve the possible causal relevance of HDL cholesterol concentrations for risk of myocardial infarction.

It is our strong opinion that we cannot simply divide cholesterol fractions into “good” or “bad ones”. In the following, we will present arguments for this statement, based on recent published articles [12].

Zewinger et al. [13] studied a total of 3310 patients who were subjected to coronary angiography in the Ludwigshafen Risk and Cardiovascular Health (LURIC) Study. It was determined that serum amyloid A (SAA) concentrations predicted all-cause and cardiovascular mortality. The authors devised a method to calculate the levels of biologically “effective” HDL cholesterol based on SAA and HDL cholesterol data from the LURIC Study. They validated this model using two populations: the first with high SAA levels and very high risk for cardiovascular events (1255 participants with type 2 diabetes on hemodialysis in the German Diabetes and Dialysis Study) and the second from a population-based survey from Augsburg, Germany (KORA S4 Study). It was shown by *in vitro* studies that if HDL is SAA-supplemented, then endothelial nitric oxide (NO) production is reduced and endothelial production of reactive oxygen species is increased, leading, as a result, to the loss of the ability of the HDL to decrease adhesion of mononuclear cells to TNF-*α*-treated endothelial cells. The authors concluded that “SAA turned HDL into a proinflammatory particle” [12].

If an acute phase response or systemic inflammation are absent, the HDL proteome constitutes anti-inflammatory particles, but if an acute phase response or systemic inflammation are present, then the HDL proteome is remodeled to constitute particles that increase the inflammatory response. This type of system possibly evolved to provide protection against viral and bacterial infections in the past, when humans did not have long enough lifespans to suffer from chronic inflammatory diseases such as atherosclerosis or rheumatoid arthritis. The study of Zewinger et al. [13] suggests that therapies that modulate this aspect of the innate immune system may have the potential to improve the outcomes of these chronic inflammatory diseases.

The main protein in LDL is apoB (apolipoprotein B), a protein that does not exchange between particles. The main protein in HDL is apoA-I (apolipoprotein A-I), which can be exchanged between particles. It is well known that all proteins associated with HDL are continuously moving on and off the HDL particles. It was hypothesized that HDL evolved as part of the innate immune system and is a chameleon-like lipoprotein [12–16]. HDL can thus be regarded as an amplification system, enhancing inflammation in the presence of an acute phase response or in the presence of systemic inflammation, but which enhances the maintenance of an anti-inflammatory state in the absence of an acute phase response or systemic inflammation [12–16].

Moreover, evidence has been found that atherosclerosis is not the only inflammatory disease with abnormal HDL. Watanabe et al. [17] presented a study which demonstrated the association of acute phase proteins, including SAA, and complement factors with proinflammatory HDL in rheumatoid arthritis. HDL taken from subjects with rheumatoid arthritis had impaired ability to stimulate cholesterol efflux [18].

We must also mention that new clinical studies support all laboratory findings listed above. Let us note some of them:

- Ravnskov et al. [19], in a meta-analysis of 19 cohort studies including 30 cohorts with a total of 68094 elderly people, where all-cause mortality was recorded in 28 cohorts and cardiovascular (CV) mortality in 9 cohorts, found that an inverse association between all-cause mortality and LDL-C was seen in 16 cohorts (14 of these having statistical significance) representing 92% of the number of participants, where this association was recorded. For the rest, no association was found. In two cohorts, CV mortality was highest in the lowest LDL-C quartile and with statistical significance; in seven cohorts, no association was found. They concluded that high LDL-C is inversely associated with mortality in most people over 60 years. This finding is inconsistent with the cholesterol hypothesis (i.e., that cholesterol, particularly LDL-C, is inherently atherogenic). Since elderly people with high LDL-C live as long or longer than those with low LDL-C, their analysis can provide significant reasons to question the validity of the cholesterol hypothesis.
- During 2018, at the European Society Congress in Munich, Dr. Marc Allard-Ratick, from Emory University School of Medicine in Atlanta, presented a study carried out as part of the Emory Cardiovascular Biobank. The participants were 63 years old on average and about one-third were women. The conclusions were the following: (a) patients with HDL levels in the middle range of the spectrum, between 41 and 60 mg/dL of blood, had the lowest risk for heart attack or death from heart diseases. Opposite to this, (b) patients with HDL readings below 41 or above 60 faced a significantly increased risk for both health outcomes, demonstrating what the researchers called a “U-shaped” risk pattern. Scientists showed that patients with HDL levels exceeding 60 were found to have a 50 percent greater risk of heart disease death or heart attack, compared with those in the middle range. Race and gender did not appear to affect the findings. “The mechanism behind this finding remains unclear,” Dr. Marc Allard-Ratick said. During the same Congress, Dr. Gregg Fonarow, director of the Ahmanson-UCLA Cardiomyopathy Center and co-director of the UCLA Preventative Cardiology Program in Los Angeles, said that “research from UCLA established more than two decades ago that HDL cholesterol could—in certain individuals (including those with very high levels of HDL) and in certain circumstances—be dysfunctional and proinflammatory,” and contribute to narrowing of the arteries. “In others words, the so-called ‘good' cholesterol in terms of cardiovascular risk could go ‘bad' and be associated with excess risk,” added Fonarow, who was not involved in this work [20]. And, he said, one “surprising aspect of the study was that this association between high levels of HDL and increased risk of death or cardiovascular disease was seen more commonly in women compared to men.”
- Previously, Madsen et al. [21] reported results from a total of 52268 men and 64240 women which were included in two prospective population-based studies, the Copenhagen City Heart Study and the Copenhagen General Population Study. Men and women in the general population with extreme high HDL cholesterol paradoxically had high all-cause mortality.
- El Khoudary et al. [22], in an analysis, included 1380 females discovered that elevated HDL-C may not always be cardioprotective in postmenopausal women.
- Dr. Christopher Cannon, professor of medicine at Harvard Medical School and a cardiologist at Brigham and Women's Hospital, in a 2017 Harvard Heart Letter [23], raised the question “*HDL: Just a bystander?*” And he asked, “We are now realizing that HDL appears to be a marker for other factors that raise or lower the risk of a heart attack. Instead of acting as the good guy that helps lower heart disease risk, HDL may be more of a bystander.” People with low HDL levels tend to have other problems closely related to higher cardiovascular risk, such as being overweight and having diabetes. “When you see a low HDL value, it is often a middle-aged man with a big belly who has high blood sugar and high blood pressure,” says Dr. Cannon. It may be that those factors, rather than the low HDL, are behind the higher risk, he explains. Moreover, specialists spoke about the many faces of HDL. HDL, for example, comes in different shapes and sizes. Some types are spherical, while others are doughnut-shaped. Some types of HDL are great at plucking cholesterol from LDL and artery walls, while other types are indifferent to cholesterol, and some even transfer cholesterol the wrong way, into LDL and cells. Researchers are investigating this phenomenon using a test known as cholesterol efflux capacity testing. Currently available only in research settings, the test reveals how effective HDL particles are at moving cholesterol out of plaques and back to the liver. Preliminary findings suggest that high scores are linked to a lower heart disease risk, meaning the test might one day prove useful for predicting heart attack risk.

All these controversies regarding HDL are generated by expanding and deepening studies which were lately focused on these two lipoproteins (LDL and HDL). Nowadays we cannot divide the cholesterol between “good” and “bad” anymore, in a simplistic approach, because slowly the research involves more and more specialists outside medicine, as biophysicists, biologists, geneticists, etc., who focus the area of interest at a molecular and submolecular level, searching for the primary processes, before any clinical and paraclinical evidence (atherosclerotic plaques in our case); HDL cholesterol appears to be one of the most important risk factors in plaque formation, but this theory has not been proved yet, although there are multiple studies that pointed towards this possibility [21, 23]. Considering the major role of cholesterol in the physiopathology of ischemic heart disease, which is the leading cause of mortality worldwide, we consider a simplistic approach more appropriate, our aim being not to “turn upside down” the hole knowledge about atherosclerosis, but to afford a clearer approach regarding HDL-role in atherogenesis and to promote development of new therapies, which could impact the evolution of ischemic heart disease. This is the reason why we chose a mathematical approach. It offers precision in understanding HDL and LDL cholesterol as particles in different stages of formation and their role in atherogenesis. It is true that this is an innovative and atypical approach, but we could now demonstrate what once started as a supposition, and was later observed in multiple studies, the harmful effect of the “good” cholesterol. Despite the lack of extensive papers in this field, we have previously used this particular approach to explain acute thrombotic occlusion in an apparently healthy coronary artery through a similar fractal physics model [24].

Also, in a previous work [25] we have written about a possible “zero moment” of atherosclerosis initiation and we proposed a redefinition of “good” and “bad” cholesterol; we have proven the presence of the hysteresis cycle which bestows “memory” to complex fluids (for example, blood flow), and we have described the example of quasi-autonomous movement in the dynamics of atherosclerosis represented by the movement of “soliton” autostructures which consist of various arrangements of cholesterol crystals in the blood stream.

## 2. Methods

Chaoticity and nonlinearity are both structural and functional for any biostructure assimilated to a complex system [26]. Interaction between the structural units lead to mutual constraints and micro-/macroscopic, local/global, and individual/collective behaviour types. In such conjecture, the universality of laws for biostructures as a complex system becomes natural and must be reflected in mathematical procedures in the form of various theoretical models that could describe their dynamics.

Classically, commonly used models are usually founded on the otherwise unjustified supposition that variables describing the dynamics of any biostructure as a complex system are differentiable (see, for example, the kinetic models for blood dynamics [27, 28]). Thus, the success of these abovementioned models should be understood as sequential, on domains in which differentiability is still valid. The differentiable mathematical procedures are otherwise inadequate when the dynamics of any biostructure as a complex system should be solved, dynamics that involve both chaoticity and nonlinearity. However, in order to describe such dynamics but still employing differential mathematical procedures, it is necessary to explicitly introduce the scale resolution into the expression of variables and implicitly into the expression of equations that govern these dynamics. This means that any variable dependent on space and time coordinates, in a classical sense, will depend both on space and time coordinates and on scale resolution in the new mathematical sense (that of nondifferentiability). In other words, instead of operating with a variable described through a nondifferentiable function, approximations of this mathematical function, obtained by mediation at various scale resolutions, will be used. As a result, any variable designed to describe the dynamics of any biostructure as a complex system will function as the limit of a family of mathematical functions, being nondifferentiable for null scale resolution and differentiable for nonzero scale resolutions [29, 30].

This method of describing the dynamics of any biostructure as a complex system clearly implies the development of new geometrical structures and also of new mathematical models for which the motion laws, invariant to spatial and temporal transformations, are integrated with scale laws, invariant to spatial and temporal scales transformations. In our opinion, such a geometrical structure can be based on the concept of a fractal and the corresponding mathematical model can be based on the Fractal Theory of Motion [29, 31].

The fundamental assumption of this model is the one that the dynamics of any biostructure as a complex system will be described by continuous but nondifferentiable motion curves (fractal motion curves). These fractal motion curves exhibit the property of self-similarity in every point, which can be translated into a property of holography (every part reflects the hole). Basically, we are discussing about “holographic” implementations of dynamics of any biostructure as a complex system through Schrödinger type fractal “regimes” (i.e., describing the dynamics of any biostructure as a complex system by using Schrödinger type equations at various scale resolutions).

Therefore, the fundamental assumption of our mathematical model (in accordance with the Fractal Theory of Motion) is the one that the motions of blood's structural units (elements, HDL, LDL, colloids, etc.) [26] take place on continuous but nondifferentiable curves (fractal curves). The abovementioned hypothesis can be formulated as follows: between two successive collisions, the trajectory of a blood's structural unit is a straight line that becomes nondifferentiable at the impact point. Considering that the entirety of the collision impact points forms an uncountable set of points, it results that the trajectories of the blood's structural units become continuous but nondifferentiable curves (fractal curves). From such a perspective, taking into account the density of the collisions between blood's structural units (HDL-LDL collisions, HDL-colloids collisions, LDL-colloids collisions, etc.) and therefore the diversity of nondifferentiable type motion trajectories of blood's structural units, we can say that blood behaves both structural and functional as a multifractal. Such a case is not a singular one, for example, the brain behaves both structural and functional as a multifractal [32].

In this context, let us analyze the dynamical behaviour of specific structural units of the blood in the form of HDL and LDL cholesterol particles. The functionality of the above stated hypothesis implies the following [29, 31]:

- Any continuous but nondifferentiable curve of cholesterol particles (cholesterol fractal/multifractal curve) is explicitly scale resolution *δt* dependent, and, further, it diverges when the scale resolution tends to zero. In other words, a continuous and nondifferentiable curve is fractal (under the above acceptation of scale-divergence). This results is a natural consequence of the Lebesgue theorem (a continuous curve of finite length is differentiable or almost everywhere differentiable) [30]. Then, any fractal/multifractal curve exhibits the property of self-similarity in every one of its points, which can be translated into a property of holography (every part reflects the whole) [30].
- The dynamics of cholesterol particles are related to the behaviour of a set of functions during the zoom operation of the scale resolution *δt*. Then, *δt* will be identified with d*t*, i.e., *δt* ≡ d*t* and, consequently, it will be considered as an independent variable. We reserve the notation d*t* for the usual time as in the Hamiltonian cholesterol dynamics.
- The dynamics of cholesterol particles are described through fractal/multifractal variables, i.e., mathematical functions depending on both the space and time coordinates and the scale resolution since the differential time reflection invariance of any dynamical variable is broken. Then, in any point of the cholesterol fractal/multifractal curve, two derivatives of the variable field *Q*(*t*, d*t*) can be defined:

(1) $$\begin{array}{c} \frac{d_{+}Q\left(t,\text{d}t\right)}{\text{d}t}=\underset{\Delta t⟶0_{+}}{\lim}\frac{Q\left(t+\Delta t,\Delta t\right)-Q\left(t,\Delta t\right)}{\Delta t},\\ \frac{d_{-}Q\left(t,\text{d}t\right)}{\text{d}t}=\underset{\Delta t⟶0_{-}}{\lim}\frac{Q\left(t,\Delta t\right)-Q\left(t-\Delta t,\Delta t\right)}{\Delta t}. \end{array}$$

  The “+” sign corresponds to forward processes of cholesterol particles, while the “−” sign corresponds to the backwards ones. We must mention that in the differentiable case relations (1), under the form

(2) $$\begin{array}{c} \frac{d_{+}Q\left(t\right)}{\text{d}t}=\underset{\Delta t⟶0_{+}}{\lim}\frac{Q\left(t+\Delta t\right)-Q\left(t\right)}{\Delta t},\\ \frac{d_{-}Q\left(t\right)}{\text{d}t}=\underset{\Delta t⟶0_{-}}{\lim}\frac{Q\left(t\right)-Q\left(t-\Delta t\right)}{\Delta t}, \end{array}$$

  are equivalent (one passes from one definition to the other by the Δ*t*⟶−Δ*t* transformations).
(iv) The differential of the spatial coordinate field d*X*^*i*^(*t*, d*t*), by means of which we can describe the cholesterol dynamics, is expressed as the sum of the two differentials, one of them being scale resolution independent (differential part *d*_±_*x*^*i*^(*t*)) and the other one being scale resolution dependent (fractal/multifractal part *d*_±_*ξ*^*i*^(*t*)). Explicitly we will have

(3) $$\begin{array}{c} d_{\pm }X^{i}\left(t,\text{d}t\right)=d_{\pm }x^{i}\left(t\right)+d_{\pm }\xi^{i}\left(t,\text{d}t\right). \end{array}$$

(v) The nondifferentiable part of the spatial coordinate field, by means of which we can describe the fluctuations of cholesterol dynamics, satisfies the fractal/multifractal equation [30]:

(4) $$\begin{array}{c} d_{\pm }\xi^{i}\left(t,\text{d}t\right)=\lambda_{\pm }^{i}{\left(\text{d}t\right)}^{1/D_{\text{F}}}, \end{array}$$

  where *λ*_±_^*i*^ are constant coefficients through which the (multi)fractalization type describing the cholesterol dynamics is specified and *D*_F_ defines the fractal dimension of the cholesterol nondifferentiable curve.
  Any definition can be chosen for *D*_F_ (fractal dimension in Kolmogorov sense, fractal dimension in Hausdorff–Besikovici sense, etc. [29, 30]), but once selected, it will maintain a constant value in the dynamics analysis of cholesterol particles. In our opinion, cholesterol dynamics simultaneously takes place on geodesics with various fractal dimensions. The variety of these fractal dimensions of the cholesterol geodesics comes as a result of the cholesterol complex structure. Precisely, for *D*_F_ = 2, coherent-type processes are generated in cholesterol dynamics. For *D*_F_ < 2, correlative-type processes are induced, while for *D*_F_ > 2 noncorrelative-type ones can be found [30].
  Let us note that equation (4) proves to be fundamental in the construction of fractional power-laws scaling (for details, see [32]).
(vi) The differential time reflection invariance of any cholesterol dynamical variable is recovered by combining the derivatives *d*_+_/d*t* and *d*_−_/d*t* in the nondifferentiable operator:

(5) $$\begin{array}{c} \frac{\hat{d}}{\text{d}t}=\frac{1}{2}\left(\frac{d_{+}+d_{-}}{\text{d}t}\right)-\frac{i}{2}\left(\frac{d_{+}-d_{-}}{\text{d}t}\right),\;\;i=\sqrt{-1}. \end{array}$$

  This is a natural result of the prolongation procedure on the complex space of any dynamics and, particularly, cholesterol dynamics [33]. Applying now the nondifferentiable operator (5) to the spatial coordinate field, by means of which we can describe the cholesterol dynamics, yields the complex velocity field of cholesterol particles:

(6) $$\begin{array}{c} {\hat{V}}^{l}=\frac{\hat{d}X^{l}}{\text{d}t}=V_{\text{D}}^{l}-iV_{\text{F}}^{l}, \end{array}$$

  with

(7) $$\begin{array}{c} V_{\text{D}}^{l}=\frac{1}{2}\frac{d_{+}X^{l}+d_{-}X^{l}}{\text{d}t},\\ V_{\text{F}}^{l}=\frac{1}{2}\frac{d_{+}X^{l}-d_{-}X^{l}}{\text{d}t}. \end{array}$$

  The real part *V*_D_^*l*^ of the cholesterol complex velocity field is differentiable and scale resolution independent (differentiable velocity field), while the imaginary one *V*_F_^*i*^ is nondifferentiable and scale resolution dependent (fractal velocity field). We remind that, according to the above-presented facts, the scale resolution dependency is applied only to the imaginary part.
(vii) In the absence of any external constraint, an infinite number of fractal/multifractal curves (geodesics) can be found relating any pair of points, and this is true on all scales of cholesterol dynamics. Then, in the fractal/multifractal space of cholesterol, all cholesterol particles are substituted with the geodesics themselves so that any external constraint can be interpreted as a selection of geodesics. The infinity of geodesics in the bundle, their nondifferentiability, and the two values of the derivative imply a generalized statistical fluid-like description [29–31]. Then, averages, variances, covariances, correlation coefficients, etc., i.e., the entire “arsenal” specific to stochastic processes become operational in describing cholesterol dynamics. Particularly, if we consider that the average of *d*_±_*X*^*i*^is

(8) $$\begin{array}{c} \langle d_{\pm }X^{i}\rangle \equiv d_{\pm }x^{i}, \end{array}$$

  through (3), it results

(9) $$\begin{array}{c} \langle d_{\pm }\xi^{i}\rangle =0. \end{array}$$

  The previous relation (9) implies that the average of the fractal/multifractal fluctuations of cholesterol dynamics is null.
(viii) Cholesterol dynamics can be described through a covariant derivative in the following form. For this, let us consider that the cholesterol fractal/multifractal curves are immersed in a 3-dimensional space and that *X*^*i*^ is the spatial coordinate field of a point on this fractal/multifractal curve. In these conditions, any variable field *Q* admits the following Taylor expansion up to the second order:

(10) $$\begin{array}{c} d_{\pm }Q=\partial_{t}Q\text{ d}t+\partial_{i}Qd_{\pm }X^{i}+\frac{1}{2}\partial_{l}\partial_{k}Qd_{\pm }X^{l}d_{\pm }X^{k}, \end{array}$$

  where the summation over repeated indices is understood. We will keep this convention in the following.

We want to remind the fact that the *Q* variable, according to the previously mentioned facts, functions as the limit of a family of mathematical functions, being nondifferentiable for null scale resolution and differentiable for nonzero scale resolutions. As a consequence, for nonzero scale resolutions, it allows a Taylor-type expansion.

These relations are valid in any point and more for the points *X*^*i*^ on the cholesterol fractal/multifractal curve which we have selected in (10). From here, forward and backward values for cholesterol variables from (10) become

(11) $$\begin{array}{c} \langle d_{\pm }Q\rangle =\langle \partial_{t}Q\text{ d}t\rangle +\langle \partial_{i}Qd_{\pm }X^{i}\rangle +\frac{1}{2}\langle \partial_{l}\partial_{k}Qd_{\pm }X^{l}d_{\pm }X^{k}\rangle . \end{array}$$

In the following, we suppose that the average values of the all variable field *Q* and its derivatives coincide with themselves and the differentials *d*_±_*X*^*i*^and d*t* are independent. Therefore, the average of their products coincides with the product of averages. Consequently, equation (11) becomes

(12) $$\begin{array}{c} d_{\pm }Q=\partial_{t}Q\text{ d}t+\partial_{i}Q\langle d_{\pm }X^{i}\rangle +\frac{1}{2}\partial_{l}\partial_{k}Q\langle d_{\pm }X^{l}d_{\pm }X^{k}\rangle . \end{array}$$

Even the average value of *d*_±_*ξ*^*i*^ is null, for the higher order of *d*_±_*ξ*^*i*^ the situation can still be different. Let us focus on the averages 〈*d*_±_*ξ*^*l*^*d*_±_*ξ*^*k*^〉. Using (4), we obtain

(13) $$\begin{array}{c} \langle d_{\pm }\xi^{l}d_{\pm }\xi^{k}\rangle =\pm \lambda_{\pm }^{l}\lambda_{\pm }^{k}{\left(\text{d}t\right)}^{\left(2/D_{\text{F}}\right)-1}\text{d}t, \end{array}$$

where we accepted that the sign + corresponds to d*t* > 0 and the sign – corresponds to d*t* < 0.

In this condition, (12) takes the form

(14) $$\begin{array}{c} d_{\pm }Q=\partial_{t}Q\text{ d}t+\partial_{i}Q\langle d_{\pm }X^{i}\rangle +\frac{1}{2}\partial_{l}\partial_{k}Qd_{\pm }x^{l}d_{\pm }x^{k}\pm \frac{1}{2}\partial_{l}\partial_{k}Q\left[\lambda_{\pm }^{l}\lambda_{\pm }^{k}{\left(\text{d}t\right)}^{\left(2/D_{\text{F}}\right)-1}\text{d}t\right]. \end{array}$$

If we divide by d*t* and neglect the terms that contain differential factors (for details, see the method from [29, 31]), we obtain

(15) $$\begin{array}{c} \frac{d_{\pm }Q}{\text{d}t}=\partial_{t}Q+v_{\pm }^{i}\partial_{i}Q\pm \frac{1}{2}\lambda_{\pm }^{l}\lambda_{\pm }^{k}{\left(\text{d}t\right)}^{\left(2/D_{\text{F}}\right)-1}\partial_{l}\partial_{k}Q, \end{array}$$

with

(16) $$\begin{array}{c} v_{+}^{i}=\frac{d_{+}x^{i}}{\text{d}t},\;\\ v_{-}^{i}=\frac{d_{-}x^{i}}{\text{d}t}. \end{array}$$

These relations also allow us to define the operators:

(17) $$\begin{array}{c} \frac{d_{\pm }}{\text{d}t}=\partial_{t}+v_{\pm }^{i}\partial_{i}\pm \frac{1}{2}\lambda_{\pm }^{l}\lambda_{\pm }^{k}{\left(\text{d}t\right)}^{\left(2/D_{\text{F}}\right)-1}\partial_{l}\partial_{k}. \end{array}$$

Under these circumstances, taking into account (6) and (17), let us calculate $\hat{d}/\text{d}t$ from (5). It results

(18) $$\begin{array}{c} \frac{\hat{d}Q}{\text{d}t}=\partial_{t}Q+{\hat{V}}^{i}\partial_{i}Q+\frac{1}{4}{\left(\text{d}t\right)}^{\left(2/D_{\text{F}}\right)-1}D^{lk}\partial_{l}\partial_{k}Q, \end{array}$$

where

(19) $$\begin{array}{c} D^{lk}=d^{lk}-i{\bar{d}}^{lk},\\ d^{lk}=\lambda_{+}^{l}\lambda_{+}^{k}-\lambda_{-}^{l}\lambda_{-}^{k},\\ {\bar{d}}^{lk}=\lambda_{+}^{l}\lambda_{+}^{k}+\lambda_{-}^{l}\lambda_{-}^{k}. \end{array}$$

Now, from (18) and (19), the explicit form of the $\hat{d}/\text{d}t$ nondifferentiable operator becomes

(20) $$\begin{array}{c} \frac{\hat{d}}{\text{d}t}=\partial_{t}+{\hat{V}}^{i}\partial_{i}+\frac{1}{4}{\left(\text{d}t\right)}^{\left(2/D_{\text{F}}\right)-1}D^{lk}\partial_{l}\partial_{k}. \end{array}$$

In the following, this operator will be identified with the derivative of the scale covariant based on the scale covariance principle.

## 3. Results

Let us now consider the principle of scale covariance (the physics laws applied to cholesterol specific dynamics are invariant with respect to scale resolution transformations) and postulate that the passage from the classical (differentiable) biophysics to the fractal/multifractal (nondifferentiable) biophysics can be implemented by replacing the standard time derivative *d*/d*t* by the nondifferentiable operator $\hat{d}/\text{d}t$. Thus, this operator plays the role of the scale covariant derivative, namely, it is used to write the fundamental equations of cholesterols dynamics in the same form as in the classic (differentiable) case. Under these conditions, applying operator (20) to the complex velocity field (6), in the absence of any external constraint, the cholesterol geodesics (motion equations) take the following form:

(21) $$\begin{array}{c} \frac{\hat{d}{\hat{V}}^{i}}{\text{d}t}=\partial_{t}{\hat{V}}^{i}+{\hat{V}}^{l}\partial_{l}{\hat{V}}^{i}+\frac{1}{4}{\left(\text{d}t\right)}^{\left(2/D_{\text{F}}\right)-1}D^{lk}\partial_{l}\partial_{k}{\hat{V}}^{i}=0. \end{array}$$

This means that the local fractal/multifractal acceleration $\partial_{t}{\hat{V}}^{i}$, the fractal/multifractal convection ${\hat{V}}^{l}\partial_{l}{\hat{V}}^{i}$, and the fractal/multifractal dissipation $D^{lk}\partial_{l}\partial_{k}{\hat{V}}^{i}$ make their balance in any point of the cholesterol fractal/multifractal curve. Moreover, the presence of the complex coefficient of viscosity-type 4^−1^(d*t*)^(2/*D*_F_)−1^*D*^*lk*^ in the cholesterol dynamics specifies that it is a rheological medium. So, both LDL and HDL have memory, as a data, by their own structure.

If the fractalisation/multifractalisation is achieved by Markov-type stochastic processes, which involve Lévy type movements [26, 29, 30] of the cholesterol particles, then

(22) $$\begin{array}{c} \lambda_{+}^{i}\lambda_{+}^{l}=\lambda_{-}^{i}\lambda_{-}^{l}=2\lambda \delta^{il}, \end{array}$$

where *λ* is a coefficient associated to the fractal/multifractal, nonfractal/nonmultifractal transition and *δ*^*il*^ is Kronecker's pseudotensor.

In these conditions, the cholesterol geodesics take the simple form

(23) $$\begin{array}{c} \frac{\hat{d}{\hat{V}}^{i}}{\text{d}t}=\partial_{t}{\hat{V}}^{i}+{\hat{V}}^{l}\partial_{l}{\hat{V}}^{i}-i\lambda {\left(\text{d}t\right)}^{\left(2/D_{\text{F}}\right)-1}\partial^{l}\partial_{l}{\hat{V}}^{i}=0, \end{array}$$

or more, by separating motions on differential and fractal/multifractal scale resolutions

(24) $$\begin{array}{c} \frac{\hat{d}V_{\text{D}}^{i}}{\text{d}t}=\partial_{t}V_{\text{D}}^{i}+V_{\text{D}}^{l}\partial_{l}V_{\text{D}}^{i}-\left[V_{\text{F}}^{l}-\lambda {\left(\text{d}t\right)}^{\left(2/D_{\text{F}}\right)-1}\partial^{l}\right]\partial_{l}V_{\text{F}}^{i}=0,\\ \frac{\hat{d}V_{F}^{i}}{\text{d}t}=\partial_{t}V_{\text{F}}^{i}+V_{\text{D}}^{l}\partial_{l}V_{\text{F}}^{i}+\left[V_{\text{F}}^{l}-\lambda {\left(\text{d}t\right)}^{\left(2/D_{\text{F}}\right)-1}\partial^{l}\right]\partial_{l}V_{\text{D}}^{i}=0. \end{array}$$

For irrotational motions of the fractal/multifractal cholesterol particles, the complex velocity field (6) takes the form

(25) $$\begin{array}{c} {\hat{V}}^{i}=-2i\lambda {\left(\text{d}t\right)}^{\left(2/D_{\text{F}}\right)-1}\partial^{i}\ln\;\psi . \end{array}$$

Then, substituting this relation in (23), the fractal/multifractal cholesterol geodesics (for details, see method from [29, 31]) becomes

(26) $$\begin{array}{c} \lambda^{2}{\left(\text{d}t\right)}^{\left(4/D_{\text{F}}\right)-2}\partial^{l}\partial_{l}\psi +i\lambda {\left(\text{d}t\right)}^{\left(2/D_{\text{F}}\right)-1}\partial_{t}\psi =0. \end{array}$$

The fractal/multifractal cholesterol variable Φ=−2*iλ*(d*t*)^(2/*D*_F_)−1^ln Ψ defines, through (25), the complex cholesterol scalar potential of the complex velocity field, while Ψ corresponds to the fractal/multifractal cholesterol state (LDL state or HDL state). Both variables, Φ and Ψ, have no direct physical meaning, but possible “combinations” of them can acquire it, if they satisfy certain conservation laws.

Let us make explicit such a situation for *ψ*. In this purpose, we first notice that the complex conjugate of *ψ*, that is $\bar{\psi }$, satisfies through (26) the following equation:

(27) $$\begin{array}{c} \lambda^{2}{\left(\text{d}t\right)}^{\left(4/D_{\text{F}}\right)-2}\partial^{l}\partial_{l}\bar{\psi }-i\lambda {\left(\text{d}t\right)}^{\left(2/D_{\text{F}}\right)-1}\partial_{t}\bar{\psi }=0. \end{array}$$

Multiplying (26) by $\bar{\psi }$ and (27) by *ψ*, subtracting the results, and introducing the notations:

(28) $$\begin{array}{c} \rho =\psi \bar{\psi },\\ J=i\lambda {\left(\text{d}t\right)}^{\left(2/D_{\text{F}}\right)-1}\left(\psi \;\nabla \bar{\psi }-\bar{\Psi \;}\nabla \psi \right), \end{array}$$

we can obtain the conservation law of the fractal/multifractal cholesterol states density:

(29) $$\begin{array}{c} \partial_{t}\rho +\nabla J=0. \end{array}$$

In (28), *ρ* corresponds to the fractal/multifractal cholesterol state density and **J** corresponds to the fractal/multifractal cholesterol current of the fractal/multifractal cholesterol states density.

According to the aforementioned statements, let us analyze the state transfer between LDL and HDL cholesterol. Thus, when cholesterol particles are subjected to an external constraint, i.e., an inflammation which can be assimilated to a scalar potential *U*, their dynamics are described through the fractal/multifractal geodesics of the form (obtained from equation (26) for the external constraint *U* [31])

(30) $$\begin{array}{c} \lambda^{2}{\left(dt\right)}^{\left(4/D_{\text{F}}\right)-2}\partial^{l}\partial_{l}\psi +i\lambda {\left(\text{d}t\right)}^{\left(2/D_{\text{F}}\right)-1}\partial_{t}\psi -\frac{U}{2}\psi =0. \end{array}$$

In the one-dimensional case, the above equation becomes

(31) $$\begin{array}{c} \lambda^{2}{\left(\text{d}t\right)}^{\left(4/D_{\text{F}}\right)-2}\partial_{zz}\psi \left(\mathrm{z,t}\right)+i\lambda {\left(\text{d}t\right)}^{\left(2/D_{\text{F}}\right)-1}\partial_{t}\psi \left(\mathrm{z,t}\right)-\frac{U}{2}\psi \left(\mathrm{z,t}\right)=0. \end{array}$$

If the external scalar potential *U* is time independent, ∂_*t*_*U*=0, equation (31) admits the fractal/multifractal stationary solution:

(32) $$\begin{array}{c} \psi \left(z,t\right)=\theta \left(z\right)\exp\left[-\frac{i}{2m_{0}\lambda {\left(\text{d}t\right)}^{\left(2/D_{\text{F}}\right)-1}}Et\right], \end{array}$$

where *E* is the fractal/multifractal energy of the fractal/multifractal stationary cholesterol state *θ*(*x*) and *m*_0_ is the rest mass of the cholesterol particle. Then, *θ*(*x*) becomes a fractal/multifractal solution of the fractal/multifractal nontemporal equation:

(33) $$\begin{array}{c} \partial_{zz}\theta \left(z\right)+\frac{1}{2m_{0}\lambda^{2}{\left(dt\right)}^{\left(4/D_{\text{F}}\right)-2}}\left(E-U\right)\theta \left(z\right)=0. \end{array}$$

**Figure 1:**
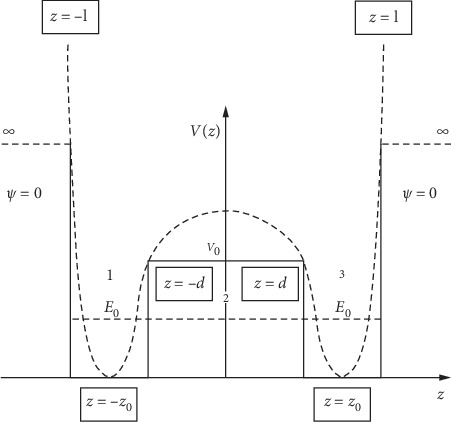
The effective potential for the case of a fractal/multifractal tunneling effect for biological systems with spontaneous symmetry breaking.

If, in such a context, we suppose that the state transfer between LDL and HDL cholesterol implies spontaneous symmetry breaking [29], then *U*=*V*(*z*) from (33) must have the form of an effective potential, as shown in Figure 1.

In these conditions, the stationary fractal/multifractal equation becomes

(34) $$\begin{array}{c} \frac{{\text{d}}^{2}\theta_{\alpha }}{\text{d}z^{2}}+\frac{1}{2m_{0}\lambda^{2}{\left(\text{d}t\right)}^{\left(4/D_{\text{F}}\right)-2}}\left[E-V_{\alpha }\right]\theta_{\alpha }=0,\;\alpha =\bar{1,3}. \end{array}$$

For each of the three regions, the solutions of the equations are

(35) $$\begin{array}{c} \theta_{1}\left(z\right)=C_{+}e^{ikz}+C_{-}e^{-ikz},\\ \theta_{2}\left(z\right)=Be^{qz}+Ce^{-qz},\\ \theta_{3}\left(z\right)=D_{+}e^{ikz}+D_{-}e^{-ikz}, \end{array}$$

with

(36) $$\begin{array}{c} k={\left[\frac{E}{2m_{0}\lambda^{2}{\left(\text{d}t\right)}^{\left(4/D_{\text{F}}\right)-2}}\right]}^{1/2},\\ q={\left[\frac{V_{0}-E}{2m_{0}\lambda^{2}{\left(\text{d}t\right)}^{\left(4/D_{\text{F}}\right)-2}}\right]}^{1/2}, \end{array}$$

and *C*_+_, *C*_−_, *B*, *C*, *D*_+_, *D*_−_ integration constants.

Due to the infinite potential in the two extreme regions, |*z*| > *l*, the fractal/multifractal state function continuity in *z*=±*l* implies

(37) $$\begin{array}{c} \theta_{2}\left(-l\right)=C_{+}e^{-ikl}+C_{-}e^{ikl}=0,\\ \theta_{3}\left(l\right)=D_{+}e^{ikl}+D_{-}e^{-ikl}=0. \end{array}$$

Since the states density |*ψ*|^2^ is not altered by the multiplication of the fractal/multifractal state function in the form of a constant phase factor, the two equations for *C*_±_ and *D*_±_ can be solved by imposing the forms

(38) $$\begin{array}{c} C_{+}=\frac{A}{2i}e^{ikl},\\ C_{-}=-\frac{A}{2i}e^{-ikl},\\ D_{+}=\frac{D}{2i}e^{-ikl},\\ D_{-}=-\frac{D}{2i}e^{ikl}, \end{array}$$

so that *θ*_1,3_ are given through simple expressions:

(39) $$\begin{array}{c} \theta_{1}\left(z\right)=A\;\sin\left[k\left(z+l\right)\right],\\ \theta_{3}\left(z\right)=D\;\sin\left[k\left(z-l\right)\right]. \end{array}$$

These, along with *ψ*_2_, lead to the concrete form of “alignment conditions” in *z*=±*d*:

(40) $$\begin{array}{c} \theta_{1}\left(-d\right)=\theta_{2}\left(-d\right),\\ \theta_{2}\left(d\right)=\theta_{3}\left(d\right),\\ \frac{\text{d}\theta_{1}}{\text{d}z}\left(-d\right)=\frac{\text{d}\theta_{2}}{\text{d}z}\left(-d\right),\\ \frac{\text{d}\theta_{2}}{\text{d}z}\left(d\right)=\frac{\text{d}\theta_{3}}{\text{d}z}\left(d\right), \end{array}$$

namely,

(41) $$\begin{array}{c} e^{-q\;d}B+e^{q\;d}C=A\;\sin\left[k\left(l-d\right)\right],\\ qe^{-q\;d}B-qe^{q\;d}C=kA\;\cos\left[k\left(l-d\right)\right]\text{ in }z=-d,=-d,\\ e^{q\;d}B+e^{-q\;d}C=-D\;\sin\left[k\left(l-d\right)\right],\\ qe^{q\;d}B-qe^{-q\;d}C=k\;D\;\cos\left[k\left(l-d\right)\right]\text{ in }z=d,=d. \end{array}$$

Due to the algebraic form of the two equation pairs, in order to establish the concrete expression of the “secular equation” (for eigenvalues *E* of the energy), Δ[*E*]=0, we avoid calculating the 4^th^ order determinant, Δ[*k*(*E*), *q*(*E*)], formed with the fractal/multifractal amplitude coefficients *A*, *B*, *C*, and *D*, by employing the following: we note with *ρ* the ratio *C*/*B* and we divide the first equation to the second one, for each pair. It results

(42) $$\begin{array}{c} \frac{e^{2q\;d}\rho +1}{e^{2q\;d}\rho -1}=-\frac{q}{k}tg\left[k\left(l-d\right)\right],\\ \frac{e^{-2q\;d}\rho +1}{e^{-2q\;d}\rho -1}=\frac{q}{k}tg\left[k\left(l-d\right)\right], \end{array}$$

which leads to the equation for *ρ*:

(43) $$\begin{array}{c} \frac{e^{2q\;d}\rho +1}{e^{2q\;d}\rho -1}+\frac{e^{-2q\;d}\rho +1}{e^{-2q\;d}\rho -1}=0. \end{array}$$

We find

(44) $$\begin{array}{c} \rho^{2}=1, \end{array}$$

which implies

(45) $$\begin{array}{c} \rho_{-}=-1,\\ \rho_{+}=1. \end{array}$$

For *ρ*_+_=1, the amplitude function, *θ*_2_(*z*)≅ coth(*qz*), is symmetric just as the fractal/multifractal states of cholesterol with regard to the (spatial) reflectivity against the origin. Then, the permitted value equation of the energy of these states, *E*_S_, has the concrete form:

(46) $$\begin{array}{c} \tan\left[k_{\text{S}}\left(l-d\right)\right]=-\frac{\coth\left(q_{\text{S}}d\right)}{q_{\text{S}}}k_{\text{S}}, \end{array}$$

where

(47) $$\begin{array}{c} k_{\text{S}}={\left[\frac{E_{\text{S}}}{2m_{0}\lambda^{2}{\left(\text{d}t\right)}^{\left(4/D_{\text{F}}\right)-2}}\right]}^{1/2},\\ q_{\text{S}}={\left[\frac{V_{0}-E_{\text{S}}}{2m_{0}\lambda^{2}{\left(\text{d}t\right)}^{\left(4/D_{\text{F}}\right)-2}}\right]}^{1/2}. \end{array}$$

For *ρ*_−_=−1, the amplitude function, *θ*_2_(*z*)≅ sinh(*qz*), so that the states will be antisymmetric and permitted values equation, *E*_A_, becomes

(48) $$\begin{array}{c} \tan\left[k_{\text{A}}\left(l-d\right)\right]=-\frac{\tanh\left(q_{\text{A}}d\right)}{q_{\text{A}}}k_{\text{A}}, \end{array}$$

where

(49) $$\begin{array}{c} k_{\text{A}}={\left[\frac{E_{\text{A}}}{2m_{0}\lambda^{2}{\left(\text{d}t\right)}^{\left(4/D_{\text{F}}\right)-2}}\right]}^{1/2},\\ q_{\text{A}}={\left[\frac{V_{0}-E_{\text{A}}}{2m_{0}\lambda^{2}{\left(\text{d}t\right)}^{\left(4/D_{\text{F}}\right)-2}}\right]}^{1/2}. \end{array}$$

Because both eigenvalues equations are strongly transcendent, a direct estimation of solutions *E*_S,A_ could be possible only by means of numerical methods, which in our case is not necessary.

It results, for now, at least qualitatively, that the presence of the barrier (of finite height *V*_0_) between −*d* and *d*, leads to the splitting of the fundamental level *E*_0_ into two sublevels *E*_S_, *E*_A_, accounting for the two types of fractal/multifractal states, symmetric and antisymmetric, respectively, in which the system can be found (both states can be associated to LDL and HDL). More precisely, we can see here a process of coupling between two different fractal/multifractal (LDL and HDL) states, made possible through a fractal/multifractal tunneling effect.

In the following, the previous results will be calibrated to cholesterol dynamics. Indeed, the identification of LDL and HDL states can be performed by admitting that Δ*E*=|*E*_A_ − *E*_S_| is small when compared with *E*_0_, i.e., Δ*E* ≪ *E*_0_, which implies the fact that *q*_A,S_ are very close to

(50) $$\begin{array}{c} q_{0}={\left[\frac{V_{0}-E_{0}}{2m_{0}\lambda^{2}{\left(\text{d}t\right)}^{\left(4/D_{\text{F}}\right)-2}}\right]}^{1/2}, \end{array}$$

and considering that

(51) $$\begin{array}{c} \frac{\coth\left(q_{0}d\right)}{\tanh\left(q_{0}d\right)}={\left[\frac{\exp\left(q_{0}d\right)+1}{\exp\left(q_{0}d\right)-1}\right]}^{2}>1, \end{array}$$

with *d* > 0 it results that (in accordance with the observation that the cholesterol particle velocity, in its sanguine dynamics, is inversely proportional with its minimum relevant dimension [3, 21]) the fast structure, i.e., HDL, can be associated with the antisymmetric state of energy *E*_A_, while the slower structure, i.e., LDL, can be associated with the symmetric state of energy *E*_S_. If we consider, at the same resolution scale, the functionalities of relations [29, 31]

(52) $$\begin{array}{c} E_{\text{A}}=2m_{0}\lambda {\left(\text{d}t\right)}^{\left(2/D_{\text{F}}\right)-1}\tau_{\text{A}}^{-1},\\ E_{\text{S}}=2m_{0}\lambda {\left(\text{d}t\right)}^{\left(2/D_{\text{F}}\right)-1}\tau_{\text{S}}^{-1}, \end{array}$$

where *τ*_A_ and *τ*_S_ are state transfer specific times of HDL and LDL, respectively, and admitting that this transfer takes place through coherence, which implies *D*_F_⟶2, it results that *E*_A_/*E*_S_=*τ*_S_/*τ*_A_ > 1. Therefore, the HDL to LDL state transfer takes a shorter time than the reverse process. This result is in accordance with clinical observations [16–19, 21–23, 34].

Taking the above into account, we can thus state that LDL and HDL are two different states of the same biological structure, like in the case of neutron and proton which are two different states of the same particle, named nucleon. The state transfer between LDL and HDL occurs by means of a fractal/multifractal tunneling effect.

## 4. Conclusions

The main conclusions of the present paper are the following:

- We build a mathematical model for describing cholesterol dynamics, based on recent scientific evidence that cholesterol (especially HDL and LDL) has a chameleonic behaviour.
- Our mathematical model is founded on the hypothesis that both HDL and LDL dynamics are described by means of continuous and nondifferentiable motion curves (fractal/multifractal curves). In such a context, the dynamics equations in the form of fractal/multifractal-type geodesics are obtained, and from here, in the stationary case, a fractal/multifractal tunneling effect for systems with spontaneous symmetry breaking is analyzed. If the spontaneous symmetry breaking is assimilated to an inflammation (in the form of a scalar potential), then two fractal/multifractal states through a fractal/multifractal tunneling effect can be observed. These two states can be associated to biological structures such as LDL and HDL. In minimal terms, we can observe here a coupling between fractal/multifractal states of cholesterol, generated by a fractal/multifractal tunneling effect. In this case, we have two potential local parabolic wells with minimal points at ±*z*_0_. This corresponds—as opposed to the case in which the wells are independent, *v*_0_⟶*∞*—to the splitting of the main energy level, *E*_0_, into two sublevels which are separated by Δ*E*=*E*_A_ − *E*_S_. In such a context, by calibrating the model to cholesterol dynamics, it is shown that the HDL to LDL state transfer takes a shorter time than the reverse process, result which is in accordance with clinical observations.
- The fact presented above are in accordance with the latest studies results. Thus, we can unequivocally state that the role of cholesterol fractions must be clearly redefined. Our model could offer an explanation of why high values of HDL cholesterol can be “toxic” or why, in certain conditions, LDL cholesterol can be a protective factor. We can practically discuss about different states of the same entity, HDL and LDL being expressions of a unique entity—cholesterol—with a pro or antiatherogenic effect modelled by the instant state and the alternation between the two possible sides. As a consequence, as long as cholesterol fractions maintain a continuous “fluidity,” the maximum benefit will be attained if the total cholesterol, in absolute value, is decreased. Our mathematical model only enforces the recent medical findings in the field, which are more and more frequent. At the same time, in our opinion, the present mathematical model confirms and explains the apparent paradoxes from clinical studies. Furthermore, our model can be used to analyze biological dynamics at nanoscale [35], with implications in various molecular medicine fields [36, 37].

## Data Availability

There are no data sets used in this study.
